# Intratumoral T cell depletion following neoadjuvant chemotherapy in patients with muscle-invasive bladder cancer is associated with poor clinical outcome

**DOI:** 10.1007/s00262-022-03234-0

**Published:** 2022-06-30

**Authors:** Sandra van Wilpe, Shabaz Sultan, Mark A. J. Gorris, Diederik M. Somford, Heidi V. N. Kusters-Vandevelde, Rutger H. T. Koornstra, Winald R. Gerritsen, Michiel Simons, Antoine G. van der Heijden, I. Jolanda M. de Vries, Niven Mehra

**Affiliations:** 1 Department of Medical Oncology, The Radboud University Medical Center, Nijmegen, The Netherlands; 2 Department of Tumor Immunology, Radboud Institute for Molecular Life Sciences, Radboud University Medical Center, Nijmegen, The Netherlands; 3 Oncode Institute, Nijmegen, The Netherlands; 4 Department of Urology, Canisius-Wilhelmina Hospital, Nijmegen, the Netherlands; 5 Department of Pathology, Canisius Wilhelmina Hospital, Nijmegen, The Netherlands; 6 Department of Medical Oncology, Rijnstate Hospital, Arnhem, The Netherlands; 7 Department of Pathology, Radboud Institute for Molecular Life Sciences, Radboud University Medical Center, Nijmegen, The Netherlands; 8 Department of Urology, Radboud Institute for Molecular Life Sciences, Radboud University Medical Center, Nijmegen, The Netherlands

**Keywords:** Urothelial cancer, Tumor-infiltrating lymphocytes, Platinum-based chemotherapy, Immunohistochemistry

## Abstract

**Background:**

Whereas neoadjuvant cisplatin-based chemotherapy (NAC) followed by a radical cystectomy remains the standard treatment for patients with muscle-invasive bladder cancer (MIBC), increasing evidence suggests that checkpoint inhibitors, either alone or in combination with chemotherapy, are effective in the (neo)adjuvant setting. The major aim of this study was to improve our understanding of the immune-modulating effects of NAC in MIBC.

**Methods:**

Tumor tissue of 81 patients was used, including 60 patients treated with NAC and 21 patients undergoing upfront cystectomy. Multiplex immunohistochemistry was performed to assess CD3^+^, CD3^+^CD8^+^, CD3^+^CD8^−^FoxP3^−^, CD3^+^FoxP3^+^, and CD20^+^ cells. Patients were classified into a favorable or unfavorable outcome group based on the development of a recurrence within a year.

**Results:**

The density of intratumoral CD3^+^ T cells decreased following NAC in patients with a recurrence at one year, while it remained stable in patients without a recurrence (median fold change 0.6 [95CI 0.3; 1.0] versus 1.0 [95CI 0.6; 2.2]). This decrease was mainly attributable to a decrease in CD3^+^CD8^−^FoxP3^−^ and CD3^+^FoxP3^+^ T cells and was not observed in patients with an early recurrence after upfront cystectomy. Additionally, in cystectomy tissue of patients treated with NAC, median CD3^+^ and CD3^+^CD8^+^ T cell densities were significantly lower in patients with versus patients without a recurrence (CD3: 261. cells/mm^2^ [95CI 22.4; 467.2]; CD8: 189.6 cells/mm^2^ [95CI 2.0;462.0]).

**Conclusion:**

T cell density decreases following NAC in MIBC patients with poor clinical outcome. Further research is needed to investigate whether this decrease in T cell density affects the efficacy of subsequent checkpoint inhibitors.

**Précis:**

The major aim of this study was to improve our understanding of the immune-modulating effects of NAC in patients with MIBC. We reveal a decline in intratumoral CD3^+^ T cell density following NAC in patients with an early recurrence.

**Supplementary Information:**

The online version contains supplementary material available at 10.1007/s00262-022-03234-0.

## Introduction

Platinum-based chemotherapy is a cornerstone in the treatment of localized muscle-invasive (MIBC) and metastatic bladder cancer (mBC). For the treatment of MIBC, neoadjuvant cisplatin-based chemotherapy (NAC) followed by a radical cystectomy is the standard treatment, increasing 5-year overall survival with 5 to 8% compared to radical cystectomy alone [1]. For patients with mBC, cisplatin-based chemotherapy is recommended as first-line treatment, inducing responses in approximately 50% of patients [2].

Since a few years, immune checkpoint inhibitors (ICIs) are available as an additional treatment option for patients with mBC. ICIs targeting programmed cell death protein 1 (PD-1) or its ligand (PD-L1) are used to treat cisplatin-ineligible patients with a PD-L1 positive tumor as well as patients that have progressed on first-line platinum-based chemotherapy. Additionally, maintenance therapy with PD-L1 inhibitor avelumab was recently approved for the treatment of patients that achieved a response or stable disease with first-line palliative chemotherapy [3].

Now that ICIs have been proven effective in patients with mBC, there is increasing interest in the application of ICIs for the treatment of localized MIBC. Data from phase I/II studies show encouraging results for neoadjuvant ICI therapy, i.e., complete responses and pathologic downstaging (*p* < T2N0) in up to 46 and 58% of cases, respectively [4–7]. The combination of ICIs and chemotherapy in the neoadjuvant setting has also been explored and resulted in complete responses and pathologic downstaging in up to 38% and 69% of patients in single-arm phase II trials [8–10]. In addition, the results of two phase III clinical trials investigating the efficacy of anti-PD-(L)1 in the adjuvant setting have recently been published. In both trials, approximately half of the patients had received prior NAC. Whereas no significant improvement was seen with atezolizumab [11], adjuvant treatment with nivolumab significantly improved median disease-free survival (20.8 versus 10.8 months), leading to the recent approval of nivolumab for this indication by the Food and Drug Administration [12]. Although ICIs appear to be effective in the (neo)adjuvant setting, the optimal treatment strategy is unclear. A better understanding of how cisplatin-based chemotherapy affects antitumor immunity might help to determine the most optimal treatment strategy.

Chemotherapy has historically been thought to act through direct killing of tumor cells. Nevertheless, it also induces various immunogenic effects which might impede or augment antitumor immunity. On the one hand, chemotherapy leads to general lymphodepletion [13] and may induce the release of proinflammatory molecules that promote the recruitment of immune suppressive cells to the tumor microenvironment [14], thereby potentially promoting tumor immune evasion. On the other hand, preclinical studies have shown that cisplatin and gemcitabine can contribute to antitumor immunity. In mice, cisplatin was found to increase both the number [15] and cytotoxic activity [16, 17] of tumor-infiltrating lymphocytes (TIL). In addition, cisplatin and gemcitabine can reduce the abundance of immune suppressive cells, such as regulatory T cells [16, 18–23].

Few studies have evaluated the effects of cisplatin and gemcitabine on antitumor immunity in patients with bladder cancer [24, 25]. Krantz and colleagues [25] characterized T cells in the sentinel nodes of 40 MIBC patients. In patients treated with NAC, the total T cell fraction (of all CD45^+^ cells) was significantly higher compared to chemotherapy-naïve patients, whereas the fraction of regulatory T cells was lower. An important limitation of this study is that the chemotherapy-naïve control group was ineligible for chemotherapy, precluding differentiation between a true effect of chemotherapy and a pre-existing difference between the cohorts. To learn more about chemotherapy-induced changes in TIL density, we need to study changes within individual patients.

The major aim of this study was to assess differences in TIL density between samples obtained before and after cisplatin-based NAC and to associate these changes with clinical outcome. To detect NAC-induced changes in the density of total (CD3^+^), cytotoxic (CD3^+^CD8^+^), helper (CD3^+^CD8^−^FoxP3^−^) and regulatory (CD3^+^FoxP3^+^) T cells, and B cells (CD20^+^), we performed multiplex immunohistochemistry (mIHC) in paired bladder cancer samples of patients treated with and without NAC. Samples of patients undergoing upfront radical cystectomy were included as controls to distinguish between NAC-induced changes and inherent differences between samples obtained during transurethral resection and cystectomy. Additionally, we evaluated the prognostic value of TILs in patients receiving NAC.

## Methods

### Patients and samples

For this retrospective study, we selected patients who underwent a radical cystectomy at the Radboud University Medical Center between 2014 and 2020 or at the Canisius Wilhelmina Hospital between 2011 and 2017. For inclusion in the NAC cohort, patients must have had received at least two cycles of cisplatin-based chemotherapy prior to radical cystectomy. Patient in the control group must have had undergone upfront radical cystectomy. All patients had to have predominant urothelial histology. Patients were excluded if they had signs of distant metastases at the time of surgery. Transurethral resection of the bladder tumor (TURBT) must had been conducted prior to NAC, if NAC was applied.

In patients with residual invasive disease at the time of cystectomy, tissue of both TURBT and cystectomy was used for mIHC. No matched tumor tissue was available for patients with a complete response of the bladder tumor (ypT0) or downstaging to carcinoma in situ (ypTis) following NAC. In these patients, only baseline tumor tissue obtained during TURBT was assessed. For exploratory purposes, cystectomy tissue of these patients was analyzed if pathology review provided indication for the original location of the tumor (i.e., local fibrosis or the appearance of giant cells). This study was approved by the local Radboudumc medical ethical committee (local ethics number 2020–6117).

### Immunohistochemistry

Information on pathologic tumor and nodal stage, resection margins, variant histology, lymphovascular invasion and the presence of CIS was collected for the original pathology reports. Per sample, one representative formalin-fixed paraffin-embedded tissue block containing high-grade, muscle-invasive tumor was selected for mIHC by a dedicated genitourinary pathologist. Tissue blocks were cut into 4 µm thick tissue sections.

As we were particularly interested in T cell markers CD3, CD8 and FoxP3 based on available evidence on NAC-induced changes on TIL [15–23], we decided to use a 7-marker IHC panel that was previously developed by our department and contained these markers. The mIHC stainings were performed using primary antibodies against CD3 (RM-9107, Thermo Fisher, clone Sp7), CD8 (M7103, DAKO, clone C8/144B), FoxP3 (14–4777, eBioscience, clone 236A/E7), CD20 (MS-340-S, Thermo Fisher, clone L26), CD45RO (MS-112, Thermo Fisher, clone UCHL-1), and pan cytokeratin, which functioned as tumor marker (ab86734, Abcam, clone AE1/AE3 + 5D3). DAPI was used as nuclear stain (NEL801001KT, PerkinElmer). For details on antibody order, dilutions and fluorochrome pairing, we refer to supplementary table 1. Methods for panel optimization and validation have been described elsewhere [26].

For the mIHC stainings, the fully automated BOND-RX stainer (Leica Biosystems) was used. Slides were deparaffinized using Bond Dewax Solution (AR9222, Leica). Antigen retrieval was performed in Bond Epitope Retrieval 2 solution (AR9640, Leica) at 95 °C for 20 min. Prior to application of the primary antibodies, slides were incubated in Opal antibody diluent (ARD1001EA, PerkinElmer) for 10 min to reduce nonspecific background staining. Slides were incubated in a primary antibody solution for 1 h at room temperature, followed by incubation in Opal Polymer anti-mouse/anti-rabbit HRP (NEL801001KT, PerkinElmer) and Opal TSA substrate (NEL801001KT, PerkinElmer). This process was repeated for all six markers. At last, DAPI nuclear counterstain, diluted in TBST, was applied manually for 5 min and slides were embedded in Fluormount-G (0100–01, ITK).

### Tissue imaging and analysis

**Fig. 1:**
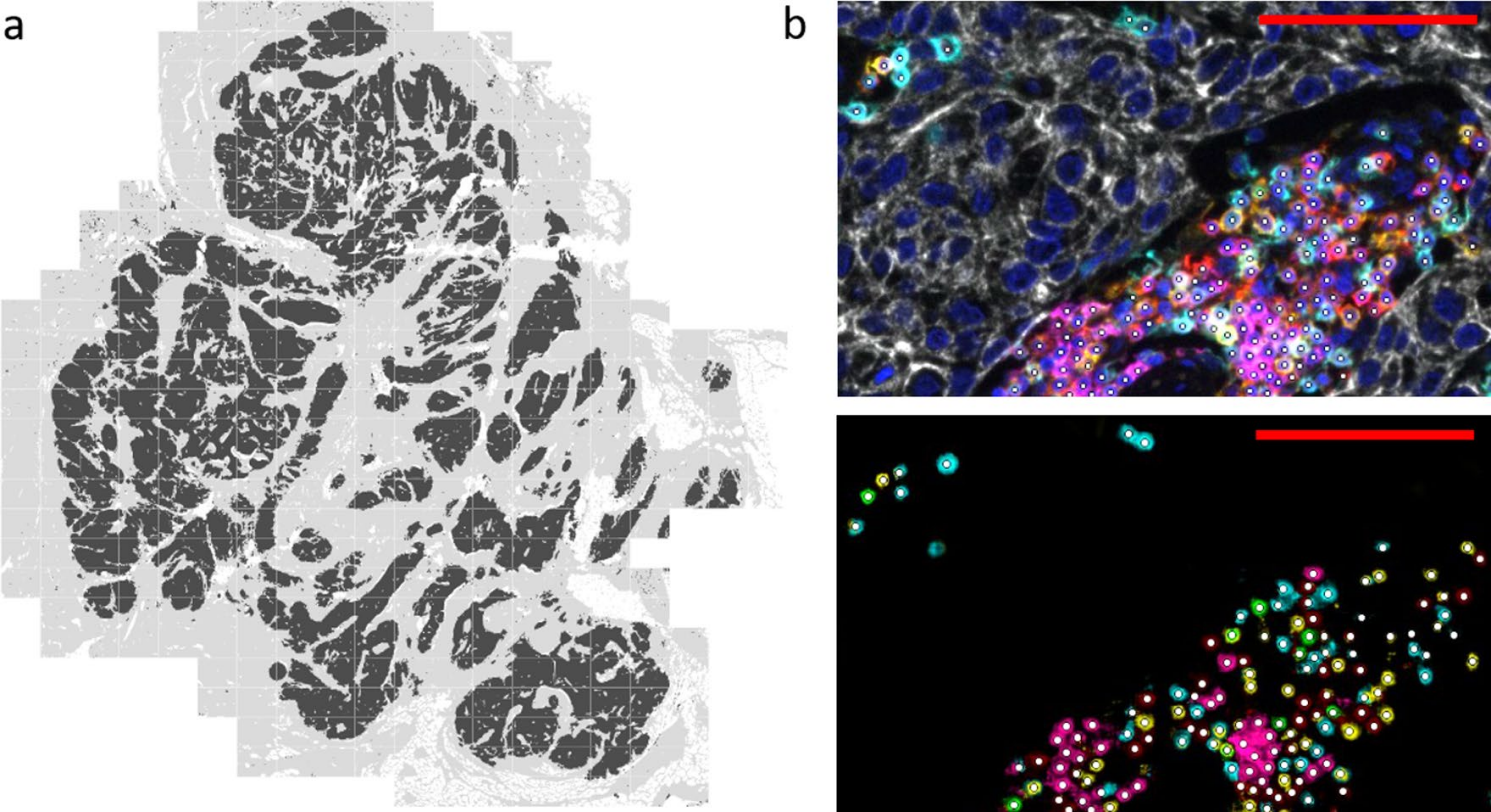
Data processing and analysis of mIHC images. **a** Tissue segmentation by PerkinElmer inForm. An algorithm was trained based on the expression of the seven mIHC markers and autofluorescence to discriminate between tumor (black), stroma (grey) and background (white). All tumor tissue plus one surrounding field of stroma (669 × 500 µm; separated in this image by white lines) was scanned at 20 × magnification to determine TIL density in tumor and stroma. **b** Cell segmentation (top) and phenotyping (bottom). A neural network was trained to identify T cells and B cells (white dots) based on the expression of the seven mIHC markers (red = CD3; cyan = CD8; green = FoxP3; magenta = CD20; yellow = CD45RO; white = tumor marker; dark blue = DAPI). The red scalebar represents a length of 100 µm

TILs were quantified using a fully automated approach. Stained slides were scanned using the PerkinElmer Vectra® 3 Automated Quantitative Pathology Imaging System (software version 3.0.4). Single stainings were used to set exposure times. Slides were first scanned at 4 × magnification. Using PerkinElmer Phenochart (version 1.0.9), tumor regions plus one surrounding field of stroma (669 × 500 µm) were selected for imaging at 20X magnification (Fig. 1). PerkinElmer inForm® image-analysis (version 2.4.2) was used for spectral unmixing, removal of autofluorescence signal and tissue segmentation. For tissue segmentation, an algorithm was trained based on the expression of the seven IHC markers and the autofluorescence signal to discriminate between tumor, stroma, and background (Fig. 1). Images and tissue phenotyping data were then exported from inForm for cell identification and phenotyping by an in-house developed neural network [27]. In short, the neural network was trained by annotating over 40.000 cells. It identifies T cells and B cells based on the expression of the seven IHC markers in our panel and predicts for each identified cell which of the markers is expressed (Fig. 1). The data generated by the neural network were exported in Flow Cytometry Standard (FCS) files. Cell populations were gated in FlowJo (version 10, Tree Star Inc., Ashland, OR, USA) using the predicted marker expression of the neural network (Supplementary Fig. 1A).

### Immune cell subsets

In this study, we differentiated between intratumoral TILs and TILs located in the tumor-surrounding stroma. We present data on 5 TIL subsets: total T cells (CD3^+^), cytotoxic T cells (CD3^+^CD8^+^), helper T cells (CD3^+^CD8^−^FoxP3^−^), regulatory T cells (CD3^+^FoxP3^+^), and B cells (CD20^+^). Unfortunately, we were not able to accurately distinguish CD45RO^+^ from CD45RO^−^ cells. Hence, this marker was ignored (Supplementary Fig. 1B).

### Clinical outcome

To relate (changes in) TIL density with clinical outcome, patients were dichotomized based on the development of disease recurrence within the first year after radical cystectomy. The date of radical cystectomy was chosen as *t* = 0. The time-point of one year was chosen as most clinically relevant interval following cystectomy. If a recurrence occurs after one year, we consider restarting chemotherapy with palliative intent. Additionally, in clinical trials comparing neoadjuvant chemotherapy plus radical cystectomy versus upfront radical cystectomy, disease-free survival curves appear to diverge primarily in the first year after cystectomy [28, 29]. Nevertheless, to exclude bias due to the chosen interval of 1 year, we also performed univariate cox proportional hazards analyses to relate fold changes in TIL density to time to recurrence.

Patients received follow-up according to standard hospital procedures. In the first year, patients were followed up every 3 months with alternately a CT scan of thorax and abdomen or a chest X-ray. In the second year, patients underwent a CT scan every 6 month and, in the third year, a CT scan once a year. All recurrences were confirmed by imaging. Patients who did not have sufficient follow-up data (*n* = 3) or died within one year without developing a recurrence (*n* = 3) were excluded from analyses comparing patients with versus without an early recurrence. In Kaplan Meier curves for disease-free survival, these patients were included, but they were censored at the time of last follow-up or non-cancer related death.

We correlated TIL density with disease recurrence rather than overall survival as the number of deaths was relatively low and only part of the patients had access to immunotherapy after the development of metastatic disease.

### Statistical analyses

To compare median TIL density, Gardner-Altman estimation plots were generated using the dabestr package (version 0.3.0, with 0.3.9999 branch bugfixes applied) [30]. Dabestr calculates 95% confidence intervals (95CI) through nonparametric bootstrap resampling, taking 5000 bootstrap resamples per analysis. Confidence intervals were bias-corrected and accelerated (BCa bootstrap). To compare the median differences between paired TURBT and cystectomy samples, paired analyses were performed. Unpaired analyses were performed to compare TIL density in TURBT or cystectomy tissue between patients with and without a disease recurrence at one year. To visualize disease-free survival in patients with and without TIL density above median, Kaplan–Meier curves were generated using the survival package. To relate fold changes to time to recurrence, univariate cox proportional hazard analyses were performed. In these analyses, *p* values were calculated using the likelihood ratio test. All analyses were performed in R version 4.0.2.

## Results

### Patient and tumor characteristics

**Fig. 2:**
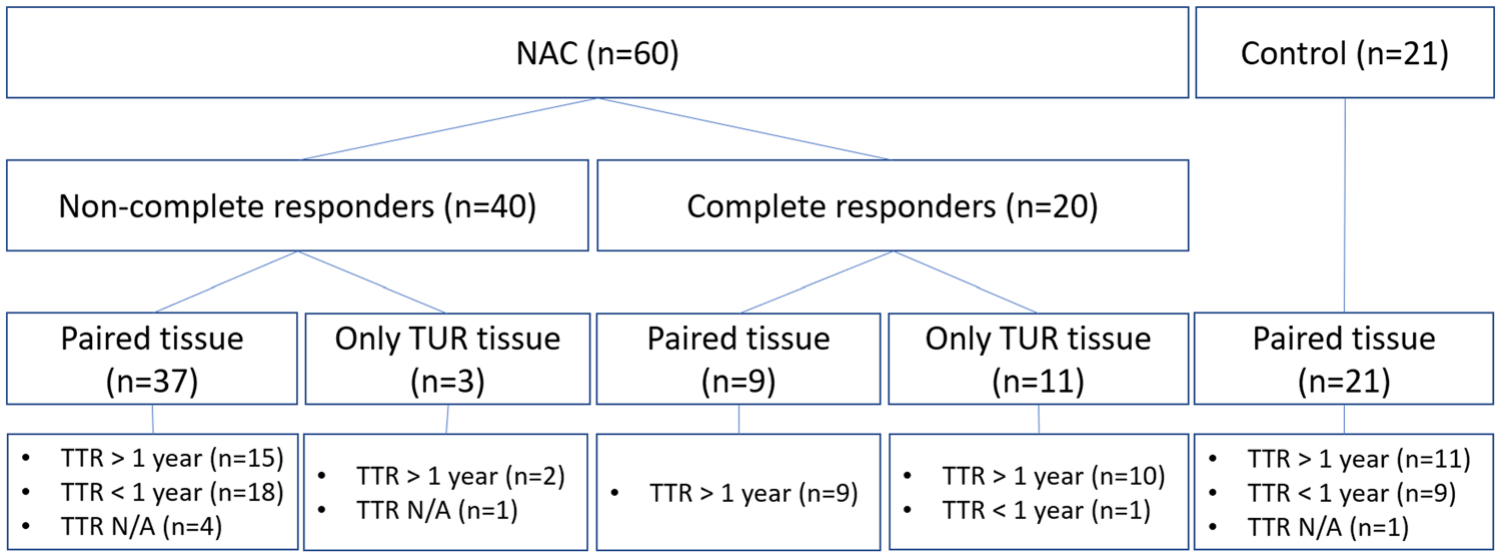
Diagram of the study population of patients with muscle-invasive bladder cancer who underwent radical cystectomy with or without neoadjuvant chemotherapy. In total, 81 patients were included. Sixty patients were treated with NAC. Of the 60 patients treated with NAC, 20 had no residual muscle-invasive disease in the bladder at the time of cystectomy (15 × ypT0, 5 × ypTis). In three additional patients with residual muscle-invasive disease, tumor tissue was no longer present in the archival tissue blocks. Thus, paired tumor samples were available for 37 patients treated with NAC. Twenty-one patients underwent upfront radical cystectomy. Paired tumor samples were available for all 21 patients. To compare (changes) in TIL density between patients with and without a favorable clinical outcome, patients were dichotomized based on whether they developed a disease recurrence within one year after radical cystectomy. Patients who did not have sufficient follow-up data (*n* = 3) or died within one year without developing a recurrence (*n* = 3) were censored. Abbreviations: NAC = Neoadjuvant chemotherapy, TTR = time to recurrence, TURBT = transurethral resection of the bladder tumor

In total, 81 patients were included (Fig. 2). Sixty patients underwent NAC, most of whom were treated with cisplatin plus gemcitabine (95%) and received four cycles of chemotherapy (70%). Median disease-free and overall survival in these patients were not reached (NR) after a median follow-up of 35 months (range: 3–119 months). Of the 60 patients undergoing NAC, 20 had no residual muscle-invasive disease in the bladder at the time of cystectomy (15 × ypT0, 5 × ypTis). In three additional patients with residual muscle-invasive disease, tumor tissue was no longer present in the archival tissue blocks. Thus, paired tumor samples were available for 37 patients treated with NAC. Twenty-one patients underwent upfront radical cystectomy (control group). In these patients, median disease-free and overall survival were 14 (8–NR) and 21 months (9–NR). Paired tumor samples were available for all 21 patients. Patient and tumor characteristics are summarized in Table 1.

**Table 1:** Patient and tumor characteristics

	**NAC (n = 60)**	**Control (n = 21)**
Age in years, median (range)	66 (42 – 79)	74 (57 – 87)
Sex, n (%)		
Male	41 (68)	14 (67)
Female	19 (32)	7 (33)
Chemotherapy regimen, n (%)		
Cisplatin/gemcitabine	57 (95)	-
Cisplatin/gemcitabine followed by carboplatin/gemcitabine	2 (3)	-
Dose dense MVAC	1 (2)	-
Number of chemotherapy cycles, n (%)		
2	5 (8)	-
3	7 (12)	-
4	42 (70)	-
5 to 6	6 (10)	-
cTNM stage at diagnosis, n (%)		
T1N0M0	0 (0)	2 (10)
T2N0M0	16 (27)	13 (62)
T3-4N0M0	19 (32)	5 (24)
N1-3M0	25 (42)	1 (5)
pTNM stage at cystectomy, n (%)		
T0/isN0M0	17 (28)	0
T2N0M0	10 (17)	9 (42)
T3-4N0M0	12 (20)	4 (19)
N1-3M0	21 (35)	8 (38)
Surgical resection margins, n (%)		
R0	54 (90)	19 (90)
R1	6 (10)	2 (10)
Presence of CIS, n (%)		
Yes	22 (37)	11 (52)
No	18 (30)	0
Unknown	20 (33)	10 (48)
Presence of lymphovascular invasion, n (%)		
Yes	12 (20)	8 (38)
No	34 (57)	3 (14)
Unknown	14 (23)	10 (48)
Histology, n (%)		
Pure urothelial carcinoma	41 (68)	15 (71)
Predominant urothelial carcinoma with variant histology	19 (32)	6 (29)
Squamous	9 (15)	2 (10)
Glandular	2 (3)	1 (5)
Nested	3 (5)	0 (0)
Other	5 (8)	3 (14)
Disease-free survival in months, median (95% CI)	NR (19 – NR)	14 (8 – NR)
Overall survival in months, median (95% CI)	NR (NR – NR)	21 (9 – NR)

*CIS* carcinoma in situ, *NAC* neoadjuvant chemotherapy, *NR* not reached

### Immune cell infiltration in paired tumor samples

First, we compared TIL density between TURBT and cystectomy tissue of 37 patients undergoing NAC and 21 patients undergoing upfront radical cystectomy (supplementary Fig. 2). As it is important to consider intraindividual changes in TIL density relative to baseline TIL density, we calculated fold changes and compared these fold changes between patients in the NAC and control group. In patients treated with NAC, intratumoral CD3^+^ and CD3^+^CD8^−^FoxP3^−^ TIL slightly decreased following NAC (median fold change CD3^+^ TIL: 0.8 [95CI 0.5; 1.0]; median fold change CD3^+^CD8^−^FoxP3^−^ TIL: 0.8 [95CI 0.5; 0.9]). This only occurred in the NAC cohort. In the control group, no clear change in intratumoral CD3^+^ or CD3^+^CD8^−^FoxP3^−^ TIL was observed. Intratumoral CD3^+^FoxP3^+^ TIL density decreased in both cohorts (median fold change NAC cohort: 0.6 [95CI 0.4; 1.1]; median fold change control group: 0.6 [95CI 0.4; 0.8]), suggesting that this change is related to the method of tissue collection or the TURBT procedure rather than to NAC. Intratumoral CD3^+^CD8^+^ TIL density did not clearly change in either of the cohorts and intratumoral CD20^+^ TIL density was generally very low, the median density of CD20^+^ TILs being 1.1 and 0.7 cells/mm^2^ in TURBT and cystectomy tissue, respectively.

In stroma, the density of all five TIL subsets was numerically lower in cystectomy compared to TURBT tissue, especially in the control group. This suggests that stromal tissue in TURBT samples differs from stroma in cystectomy specimens. In further support of this, we observed differences in the tumoral and stromal surface areas between TURBT and cystectomy tissue. TURBT samples generally contained more tumor tissue. Across all TURBT samples, the median surface areas of the tumoral and stromal compartment were 55.20 (range: 6.89–188.46) and 37.60 mm^2^ (range: 2.23 107.84), respectively. Cystectomy samples, on the other hand, contained more stromal tissue, the median surface areas of the tumoral and stromal compartment being 33.36 (0.41–146.49) and 42.22 mm^2^ (3.79–158.42). Because of this, we decided to focus on the changes in intratumoral TIL in subsequent analyses.

### Association between changes in TIL density and disease recurrence

To relate changes in TIL density following NAC with clinical outcome, we next divided the patients into a group that developed a recurrence within the first year after cystectomy (*n* = 18) and a group that did not develop a recurrence within this interval (*n* = 15). Tumor stage at cystectomy tended to be higher in patients who developed a recurrence within one year compared to patients who did not develop a recurrence (pT2: 22% vs. 47%, pT3: 44% vs. 47%, pT4: 33% vs. 7%). Additionally, patients with an early recurrence more often had nodal disease at cystectomy (67% vs. 20%) and positive surgical margins (22% vs. 7%).

**Fig. 3:**
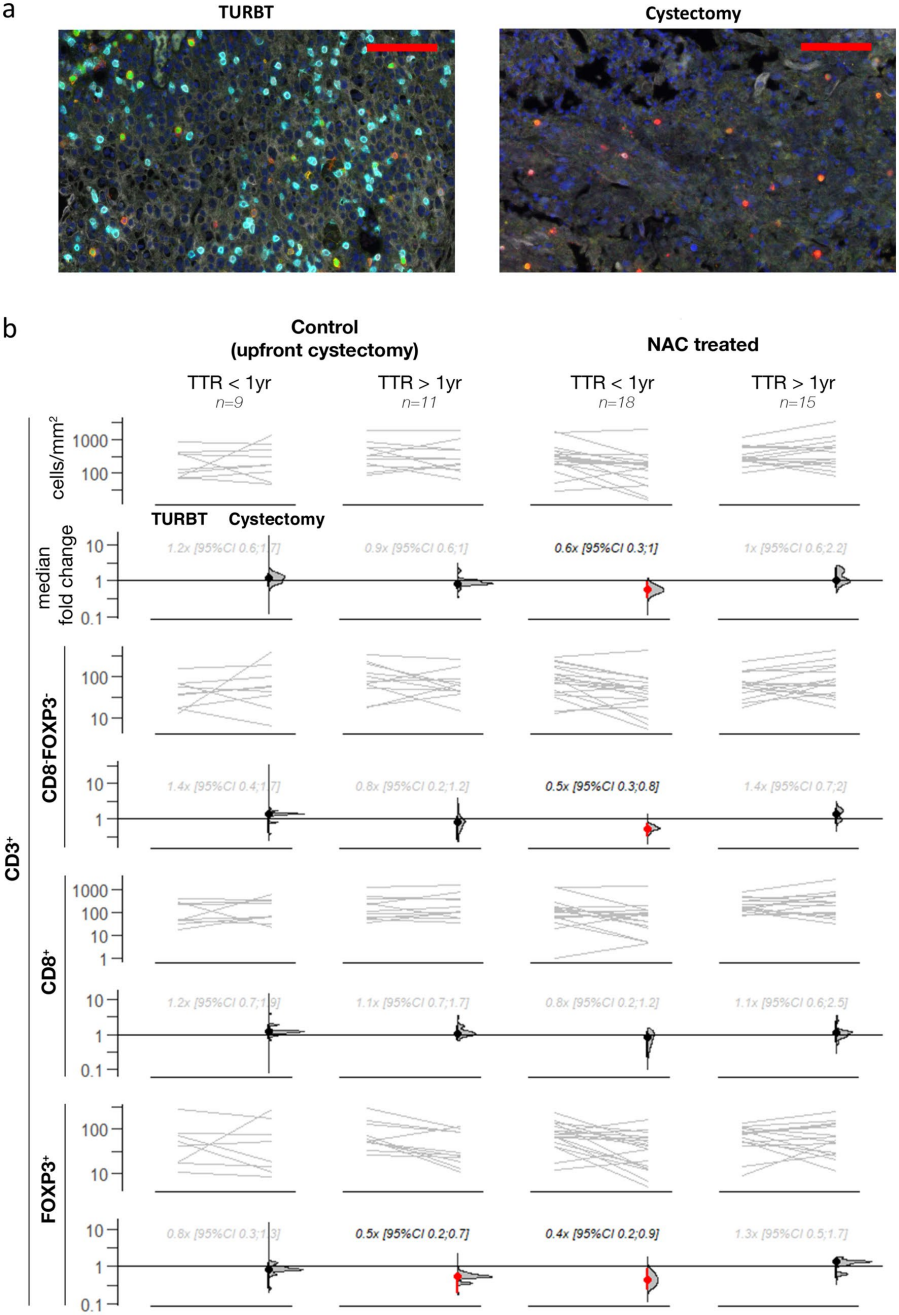
Association between changes in TIL density and disease recurrence in patients with muscle-invasive bladder cancer who underwent radical cystectomy with or without neoadjuvant chemotherapy. (a) A TURBT and cystectomy specimen of a patient showing a strong decrease in TIL density following NAC and a recurrence within the first year after radical cystectomy. (b) Gardner-Altman estimation plots depicting differences in the density of total CD3^+^, CD3^+^ CD8^−^FoxP3^−^, CD3^+^ CD8^+^ and CD3^+^ FoxP3^+^, between TURBT and cystectomy tissue of patients treated with upfront radical cystectomy (left) and NAC (right). Both cohorts were subdivided based on the presence of a disease recurrence at one year. For each TIL cell type, the upper subplot show the absolute TIL densities in TURBT and cystectomy tissue. In the lower subplot, the median fold changes are depicted as bootstrap sampling distributions. The 95% confidence intervals are indicated by the ends of the vertical error bars. Red error bars indicate a 95% confidence interval not crossing 1. Abbreviations: NAC = Neoadjuvant chemotherapy, TTR = time to recurrence, TURBT = transurethral resection of the bladder tumor

Whereas intratumoral TIL density was stable or slightly increased in patients who were disease-free at one year, patients that developed a recurrence had a decrease in intratumoral CD3^+^, CD3^+^CD8^−^FoxP3^−^, and CD3^+^FoxP3^+^ TIL density following NAC (Fig. 3). Median fold changes for CD3^+^ TIL density were 0.6 [95CI 0.3; 1.0] in patients developing a recurrence compared to 1.0 [95CI 0.6; 2.2] in patients without a recurrence in the first year. Fold changes for CD3^+^CD8^−^FoxP3^−^ and CD3^+^FoxP3^+^ TIL differed even stronger. Median fold changes for CD3^+^CD8^−^FoxP3^−^ TIL were 0.5 [95CI 0.3; 0.8] and 1.4 [95CI 0.7; 2.0] in patients with and without a recurrence, respectively. Median fold changes for CD3^+^FoxP3^+^ TIL were 0.4 [95CI 0.2; 0.9] versus 1.3 [95CI 0.5; 1.7]. The decrease in TIL density in patients with an early recurrence appeared to be related to NAC as TIL density did not decrease in patients with an early recurrence after upfront cystectomy. In univariate cox proportional hazards analyses, the fold changes in CD3^+^ and CD3^+^CD8^−^FoxP3^−^ TIL were also significantly related to the time to disease recurrence (Supplementary Table 2).

In our cohort, seven patients received fewer or more than four cycles of NAC and these patients were overrepresented in the group that developed a recurrence within one year. However, we obtained similar findings when we repeated our analyses in patients receiving four cycles of NAC (12 patients with and 14 patients without a recurrence within 1 year). In patients with an early relapse, a clear decrease was observed in CD3^+^, CD3^+^CD8^−^FoxP3^−^ and CD3^+^FoxP3^+^ TILs, whereas TIL density remained relatively unchanged in patients that were disease-free at one year (Supplementary Fig. 3).

For exploratory purposes, lymphocyte density was also assessed in cystectomy tissue of nine patients with a complete response (ypT0) or downstaging to ypTis. Although there was no residual invasive tumor in these samples, we wondered whether there might be signs of profound immune cell infiltration at the original location of the tumor in these exceptional responders. Lymphocyte density was evaluated at locations that were deemed likely by an experienced genitourinary pathologist to have contained urothelial cancer prior to NAC. We observed that TIL density was considerably lower in these regions compared to the tumor-surrounding stroma in TURBT samples of these patients (Supplementary Fig. 4).

### Prognostic value of intratumoral lymphocytes in TURBT and cystectomy tissue

**Fig. 4:**
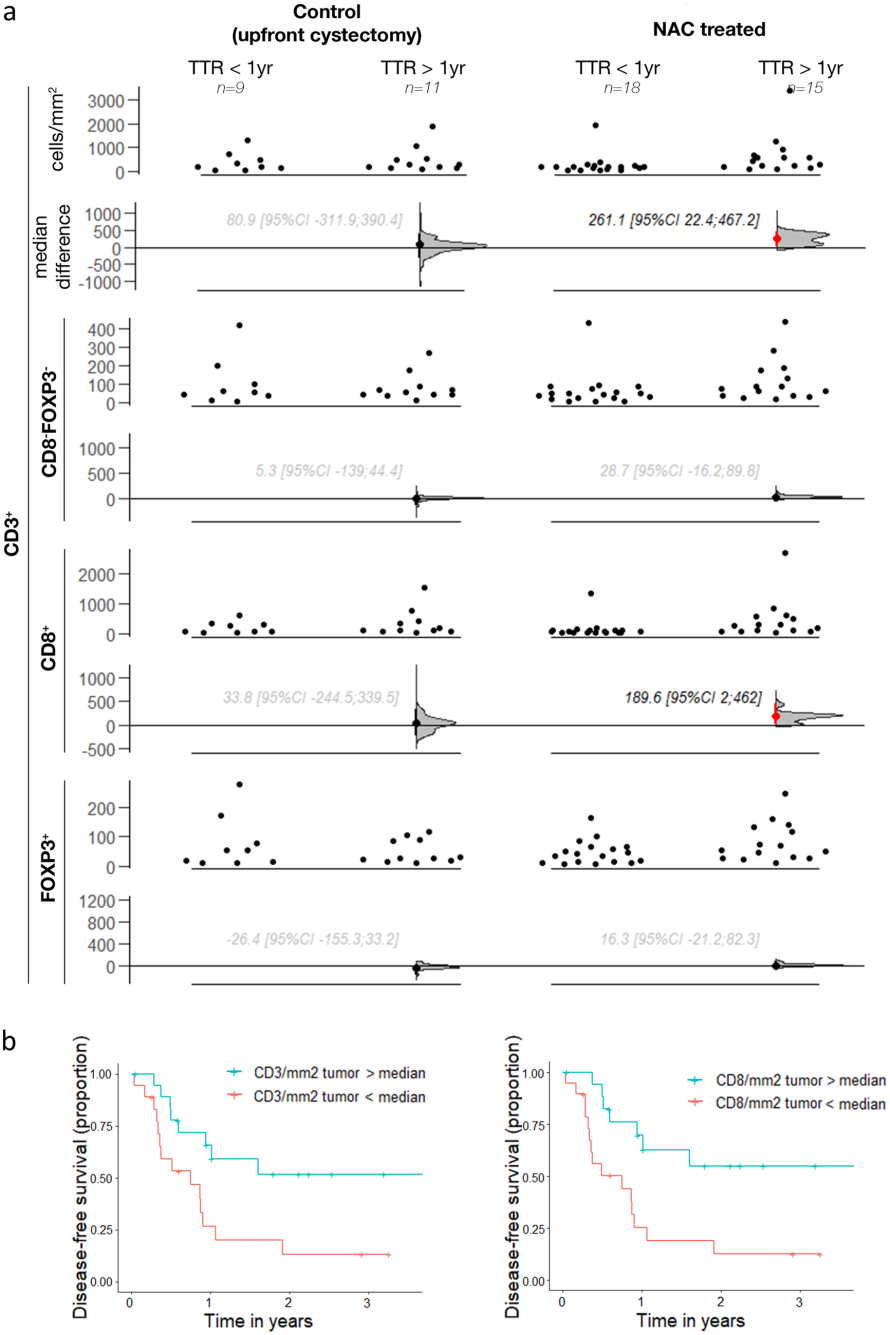
Prognostic value of intratumoral TIL density in cystectomy specimens of patients with muscle-invasive bladder cancer. (a) Gardner-Altman estimation plots showing the differences in intratumoral density of total CD3^+^, CD3^+^ CD8^−^FoxP3^−^, CD3^+^ CD8^+^ and CD3^+^ FoxP3^+^ between patients with and without a disease recurrence at one year. For each TIL cell type, the upper subplot show the absolute densities. In the lower subplot, the median differences are depicted as bootstrap sampling distributions. The 95% confidence intervals are indicated by the ends of the vertical error bars. Red error bars indicate a 95% confidence interval not crossing 0. (b) Kaplan Meier curves showing differences in disease-free survival between patients with TIL counts above versus below median following NAC (n = 37)

Although there is data on the prognostic value of T cells in MIBC patients undergoing upfront radical cystectomy [31], the relationship between TIL density and disease recurrence in MIBC patients undergoing NAC is unexplored. Therefore, we investigated the prognostic value of intratumoral TIL density in TURBT and cystectomy tissue of patients treated with NAC. TIL density in TURBT tissue was not distinctly associated with the development of a recurrence within the first year after radical cystectomy (supplementary Fig. 5 and 6). However, in cystectomy tissue of patients treated with NAC, high densities of intratumoral CD3^+^ and CD3^+^CD8^+^ TIL were associated with a time to recurrence of more than 1 year. The median difference in CD3^+^ TIL density between patients with and without a recurrence in the first year after cystectomy was 261.1 cells/mm^2^ [95CI 22.4; 467.2]; the median difference in CD3^+^CD8^+^ TIL was 189.6 cells/mm^2^ [95CI 2.0; 462.0] (Fig. 4). This association was not observed in patients undergoing upfront radical cystectomy (median difference in CD3^+^ TIL: 80.9 cells/mm^2^ [95CI − 311.9; 390.4]; median difference in CD3^+^CD8^+^ TIL: 33.8 cells/mm2 [95CI − 244.5; 339.5]).

## Discussion

The aims of this study were to improve our understanding of the immune-modulating effects of cisplatin-based chemotherapy in urothelial cancer and to evaluate the prognostic value of TILs in chemotherapy-treated patients. Utilizing paired samples, we reveal a decline in intratumoral CD3^+^ TIL density, particularly the density of CD3^+^CD8^−^FoxP3^−^ and CD3^+^FoxP3^+^ TILs, in patients with an early recurrence following NAC, while TIL density remains relatively unchanged in patients without an early recurrence. Additionally, we show that low densities of CD3^+^ and CD3^+^CD8^+^ TILs in chemotherapy-treated MIBC are related to the development of a recurrence within the first year after cystectomy.

In bladder cancer, data on NAC-induced changes in TIL density have so far been limited to one study. In this study, which did not evaluate the relationship with clinical outcome, transcriptome data were used to impute TIL fractions in pre- and post-chemotherapy samples of 20 MIBC patients [24]. No significant change in cytotoxic or regulatory T cell fractions was observed following chemotherapy. We did observe a decrease in TIL density following NAC, particularly in CD3^+^CD8^−^FoxP3^−^ TIL, but this decrease was small, i.e. a fold change of 0.8.

Our data show that changes in TIL density following NAC differ between patients with and without a recurrence in the first year. Research in other tumor types supports a relationship between chemotherapy-induced changes in the tumor immune microenvironment and clinical outcome. For example, a study in breast cancer showed that chemotherapy-induced upregulation of T cell-related gene sets is associated with favorable recurrence-free and overall survival in triple-negative breast cancer [32]. Nevertheless, this association was not observed in other breast cancer subtypes [32], suggesting that the immune-related effects of chemotherapy and its clinical implications may differ per tumor (sub)type.

There are several possible explanations why TIL density decreases following NAC in patients with an early recurrence. Chemotherapy induces systemic lymphodepletion, which might result in a lower density of TILs in the absence of a local immune response. Preclinical experiments have shown that cisplatin can increase the number and cytotoxic activity of TILs [15–17]. Patients who develop a recurrence within one year may lack this local immune response due to a lower sensitivity of these tumors to NAC or due to a poorer general immune status.

Besides the changes in TIL density following NAC, we also investigated the prognostic value of TIL density in TURBT and cystectomy tissue of patients treated with NAC. In cystectomy tissue of patients treated with NAC, high densities of intratumoral CD3^+^ and CD3^+^CD8^+^ TIL were associated with fewer early recurrences. Our findings are in line with previous reports on the prognostic value of CD3^+^ and CD3^+^CD8^+^ TIL in MIBC [31]. However, these studies either combined chemotherapy-naïve and chemotherapy-treated patients or included only chemotherapy-naïve patients. To the best of our knowledge, this is the largest cohort investigating the relationship between TIL density and disease recurrence in MIBC patients treated with NAC so far.

This study provides first insights into NAC-induced changes in TIL density in MIBC and the prognostic value of TIL in chemotherapy-treated MIBC patients. Whereas prognostic information is of high value in the clinic and could contribute to a more personalized follow-up, the implications of our findings for subsequent ICI therapy are not clear. In general, it is likely that patients with poor prognostic factors might particularly benefit from adjuvant anti-PD-(L)1. For example, it was recently suggested that adjuvant atezolizumab, which does not improve survival in unselected MIBC patients, might improve survival in patients who are positive for circulating tumor DNA (ctDNA) at the start of adjuvant therapy, the presence of ctDNA after cystectomy being a poor prognostic factor [33]. In this paper, we show that patients with a decrease in TIL density following NAC and low CD3^+^ and CD3^+^CD8^+^ T cell counts after NAC have poorer outcomes. As anti-PD-(L)1 is generally thought to be more effective in patients with high TIL density [5, 34–36], it appears counterintuitive to treat exactly these patients with anti-PD-(L)1. It was recently suggested that response to combination ICI therapy is independent of baseline TIL density [6]. This raises the question whether patients with a decline in TIL following NAC and/or low CD3^+^ and CD3^+^CD8^+^ TIL density at the time of cystectomy might particularly benefit from adjuvant combination ICI therapy. Further research is needed to find out whether (changes in) TIL density in NAC-treated MIBC patients could play a role in guiding treatment decisions.

This study has some limitations. First of all, we did not differentiate between tumor core and invasive margin [37]. The reason for this is that it is often unclear where the border of the tumor is, especially in TURBT tissue, where the original architecture is destroyed (supplementary Fig. 7). Secondly, we cannot fully exclude that the decline in TIL density in patients with an early relapse is related to changes in tumor size or stage. In our NAC cohort, tumor stage at cystectomy tended to be higher in patients who developed a recurrence within one year. However, neither in our own data nor in literature did we find strong evidence for a relation between tumor stage and/or size and TIL density (supplementary Fig. 8) [31]. Finally, we do not provide insight into early changes in TIL density during NAC. A recent study in patients with breast cancer suggests that chemotherapy might lead to an increase in TIL density after one cycle of chemotherapy, but a decrease at the end of treatment. In light of the presumed relationship between TIL density and response to anti-PD-(L)1 monotherapy, it is important to learn more about the dynamics of TIL density during NAC and find out whether a short course of chemotherapy (less than 4 cycles) followed by ICIs might be more beneficial [38].

In conclusion, this study provides first insights into NAC-induced changes in TIL density and the prognostic value of TIL in chemotherapy-treated MIBC patients. Our findings indicate that TIL density, especially the density of CD3^+^CD8^−^FoxP3^−^ and CD3^+^FoxP3^+^ TIL, decreases following NAC in patients who develop a recurrence within the first year after radical cystectomy. Additionally, we show that high CD3^+^ and CD3^+^CD8^+^ T cell counts after NAC are associated with favorable outcome. Further research into the temporal dynamics of TILs following chemotherapy and the relationship between NAC-induced changes in TIL density and response to adjuvant ICI therapy is needed to determine the optimal use and timing of immunotherapy in the (neo)adjuvant setting.

## Supplementary Information

Below is the link to the electronic supplementary material.

Supplementary file1 (PDF 1304 KB)
