# Supplementary material for: Intratumoral T cell depletion following neoadjuvant chemotherapy in patients with muscle-invasive bladder cancer is associated with poor clinical outcome
Source: Cancer Immunol Immunother. 2022 Jun 30;72(1):137–49. doi: 10.1007/s00262-022-03234-0 (PMC9813168; doi:10.1007/s00262-022-03234-0)
Supplement: Supplementary file 1 — Supplementary file1 (PDF 1304 KB) [file 262_2022_3234_MOESM1_ESM.pdf]

Supplementary figures and tables

Supplementary table 1. 7-color mIHC panel

Order	Marker	Clone	Dilution	Opal	Dilution
1	CD45RO	UCHL-1	1:3000	620	1:50
2	CD8	C8/144B	1:200	690	1:50
3	CD20	L26	1:600	570	1:50
4	CD3	Sp7	1:200	520	1:50
5	FoxP3	236A/E7	1:100	540	1:50
6	Pan cytokeratin	AE1/AE3 +5D3	1:1500	650	1:200

Supplementary table 2. Univariate cox regression analyses to assess the relationship between fold changes in TIL density and time to recurrence in patients with muscle-invasive bladder cancer undergoing a radical cystectomy with or without neoadjuvant chemotherapy

	Cell type	Hazard ratio	p-value
**NAC cohort**	CD3	0.5624	**0.03899**
	CD8	0.8706	0.398
	CD8-FoxP3-	0.5735	**0.03831**
	FoxP3	0.6142	0.06331
**Control cohort (upfront radical cystectomy)**	CD3	1.12964	0.1657
	CD8	1.14329	0.231
	CD8-FoxP3-	1.07336	0.1199
	FoxP3	1.1623	0.145

a

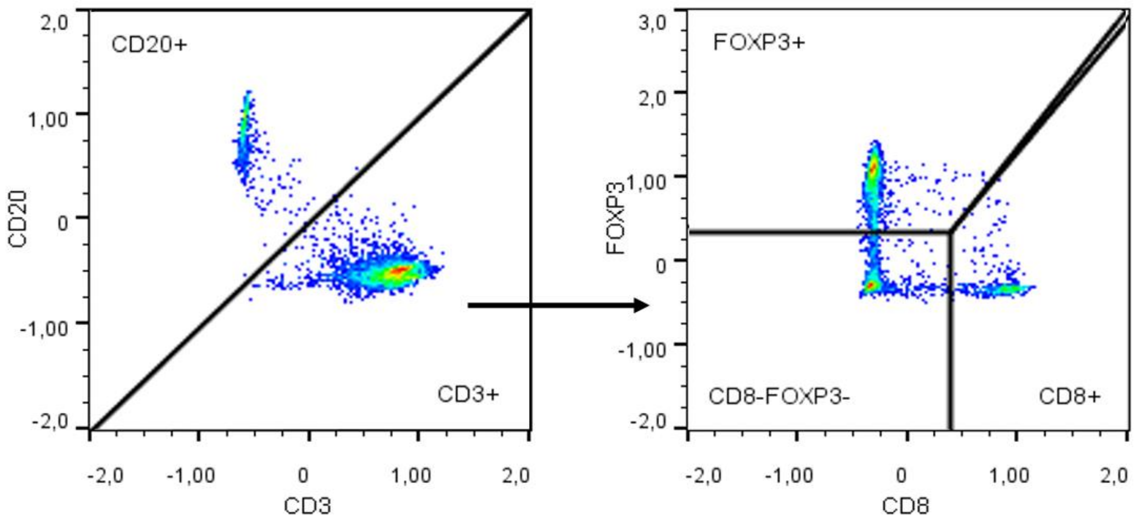

b

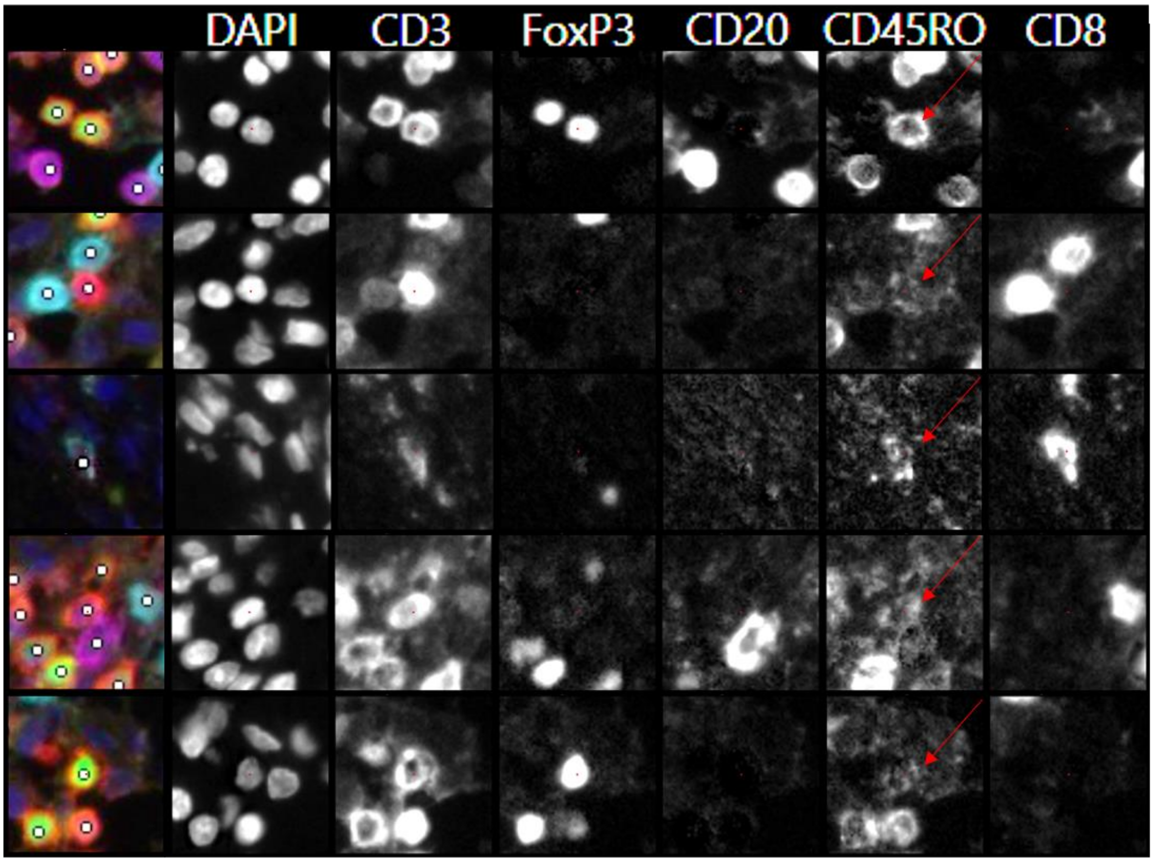

Supplementary figure 1: Cell gating in FlowJo based on the neural network's phenotype prediction and observed difficulties with CD45RO. (**a**) Stromal and intratumoral TILs recognized by the neural network were gated into $CD3^{+}$ and $CD20^{+}$ TIL (left). $CD3^{+}$ TIL were then further gated into $CD3^{+}CD8^{+}$ T cells, $CD3^{+}CD8^{-}FoxP3^{-}$ and $CD3^{+}FoxP3^{+}$ T cells (right). (**b**) When training the neural network, we generally found it easy to decide positivity for CD3, CD8, FOXP3 and CD20, whereas the status of CD45RO was more difficult to assess. Although the network agreed with the annotator in the vast majority of cases where the annotator was certain (e.g., the upper panel), the expression of CD45RO was uncertain in many other cases (lower 4 panels).

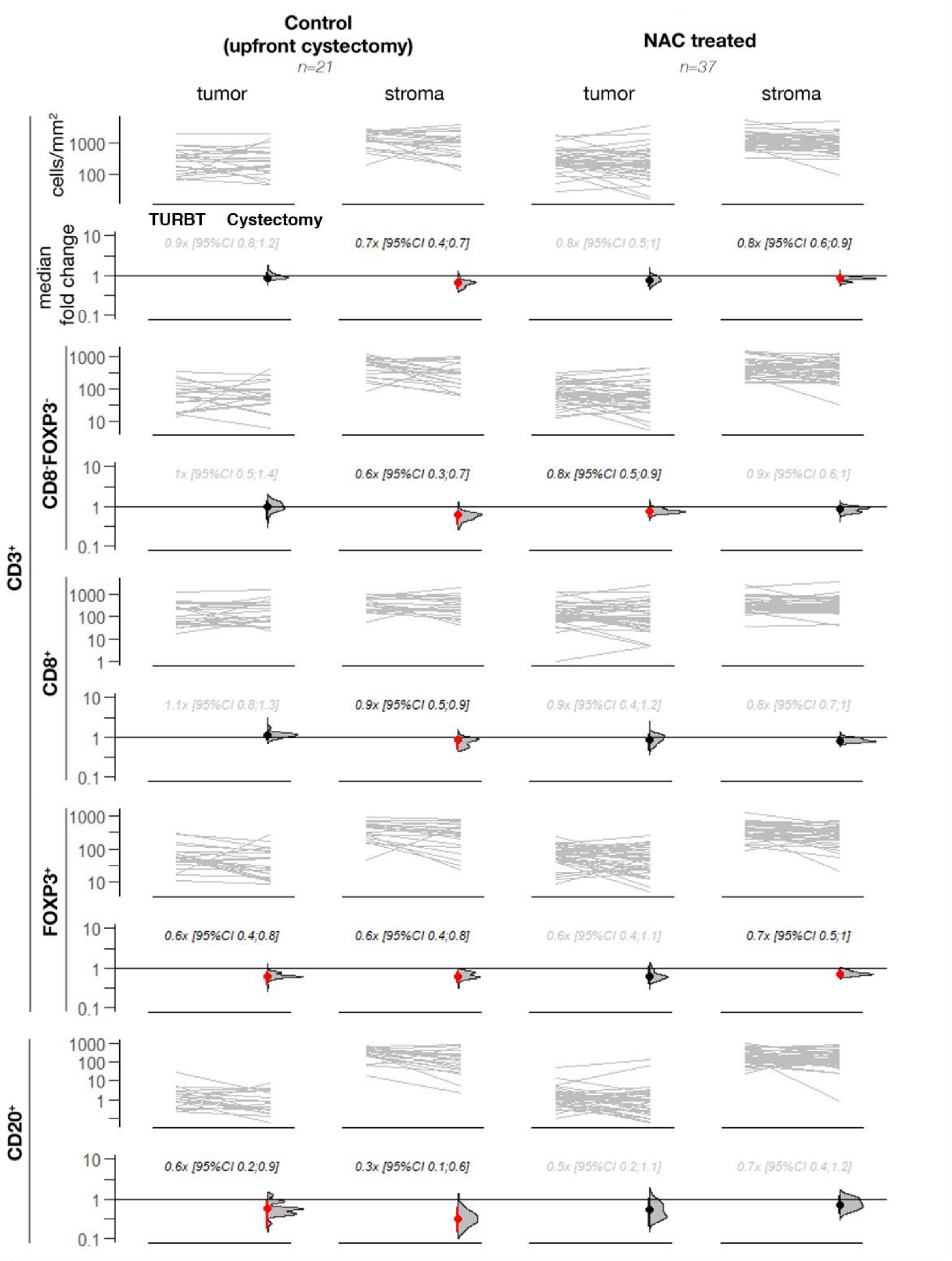

Supplementary figure 2. Differences in TIL density between paired TURBT and cystectomy samples in patients with muscle-invasive bladder cancer who underwent radical cystectomy with or without neoadjuvant chemotherapy. Gardner-Altman estimation plots showing the differences in intratumoral and stromal TIL density between paired TURBT and cystectomy samples of patients treated with upfront radical cystectomy (left) and NAC (right). The upper plots show the absolute densities in TURBT and cystectomy tissue. In a few patients no intratumoral $CD20^+$ TIL were observed. To enable visualization of cell densities on a

log scale, the CD20$^+$ densities of these patients was replaced by 0.05 cells/mm^2 (~lowest value in this plot). In the lower plots, the median fold changes are depicted as a bootstrap sampling distribution. The 95% confidence intervals are indicated by the ends of the vertical error bars. Red error bars indicate a 95% confidence interval that does not crosses 1. Abbreviations: NAC = neoadjuvant chemotherapy, TTR = time to recurrence, TURBT = transurethral resection of the bladder tumor.

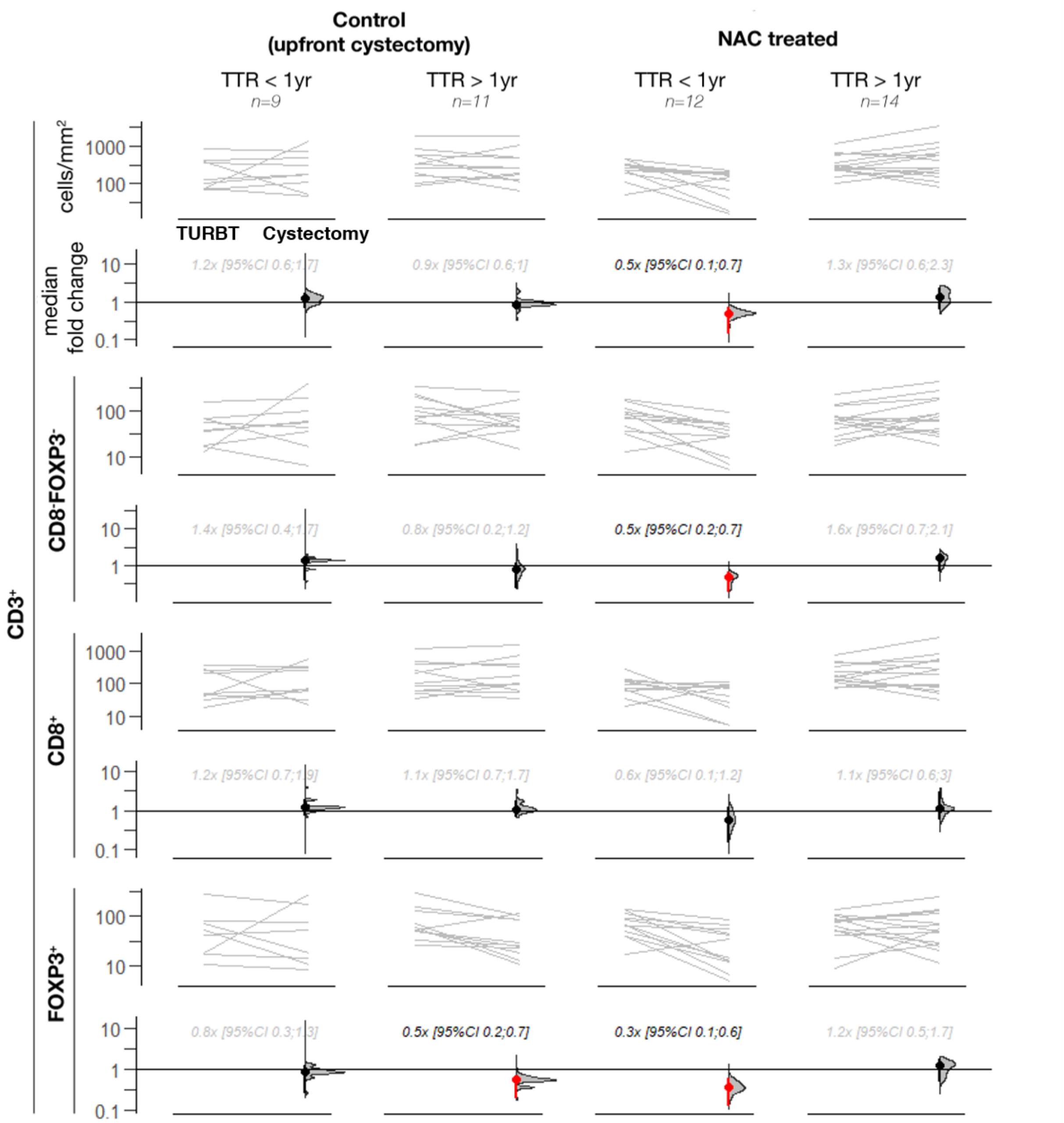

Supplementary figure 3. Fold change in intratumoral TIL density in patients with muscle-invasive bladder cancer who received 4 cycles of neoadjuvant chemotherapy prior to radical cystectomy. Gardner-Altman estimation plots showing the differences in intratumoral TIL density between TURBT and cystectomy tissue of patients treated with upfront radical cystectomy (left) and NAC (right). Both cohorts were subdivided based on the presence of a disease recurrence at one year. The upper plots show the absolute TIL densities in TURBT and cystectomy tissue. In the lower plots, the median fold changes are depicted as bootstrap sampling distributions. The 95% confidence intervals are indicated by the ends of the vertical error bars. Red error bars indicate a 95% confidence interval that does not crosses 1. Abbreviations: NAC = neoadjuvant chemotherapy, TTR = time to recurrence.

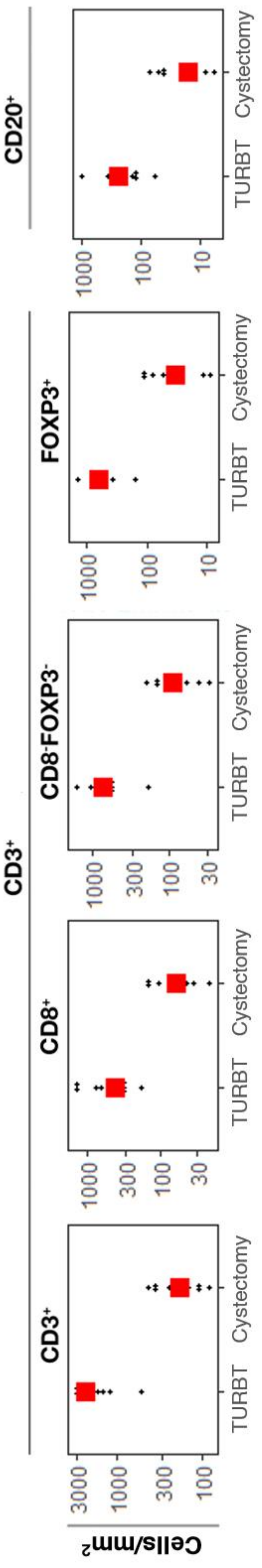

Supplementary figure 4. Differences in stromal TIL density between TURBT and cystectomy tissue of patients with muscle-invasive bladder cancer and a complete response to neoadjuvant chemotherapy. Cystectomy tissue of patients with a complete response in the bladder (pT0) or downstaging to ypTis after NAC was analyzed if pathological evaluation provided indications for the original location of the tumor (i.e., local fibrosis or the appearance of giant cells). The red square indicates the median density. Abbreviations: TURBT = transurethral resection of the bladder tumor.

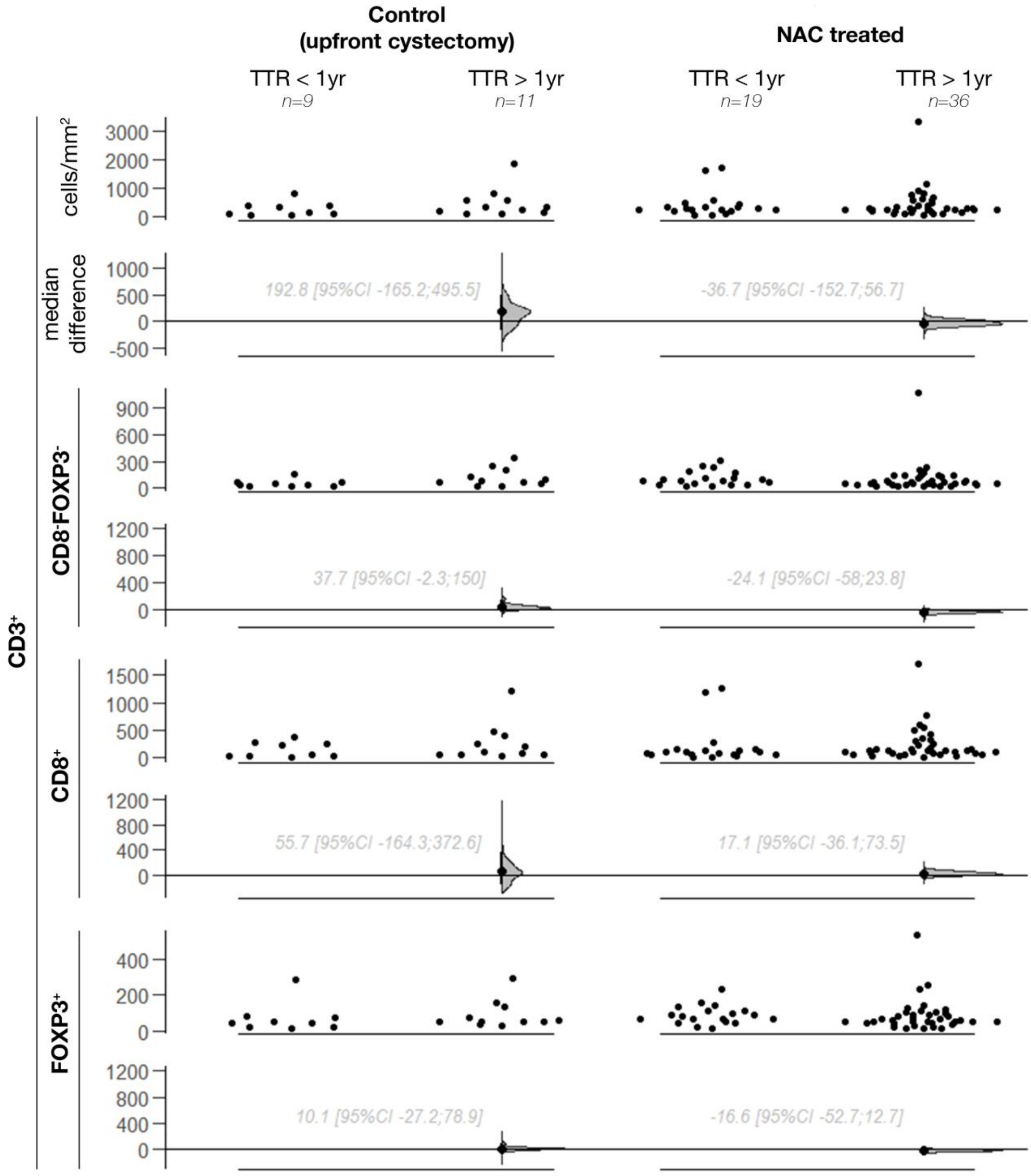

Supplementary figure 5. Prognostic value of intratumoral TIL density in TURBT tissue of patients with muscle-invasive bladder cancer undergoing radical cystectomy with or without neoadjuvant chemotherapy. Gardner-Altman estimation plots showing the differences in intratumoral TIL density between patients with and without a disease recurrence within one year. The upper plots show the absolute densities. In the lower plots, the median differences are depicted as a bootstrap sampling distribution. The 95% confidence intervals are indicated by the ends of the vertical error bars. Abbreviations: NAC = neoadjuvant chemotherapy, TTR = time to recurrence, TURBT = transurethral resection of the bladder tumor.

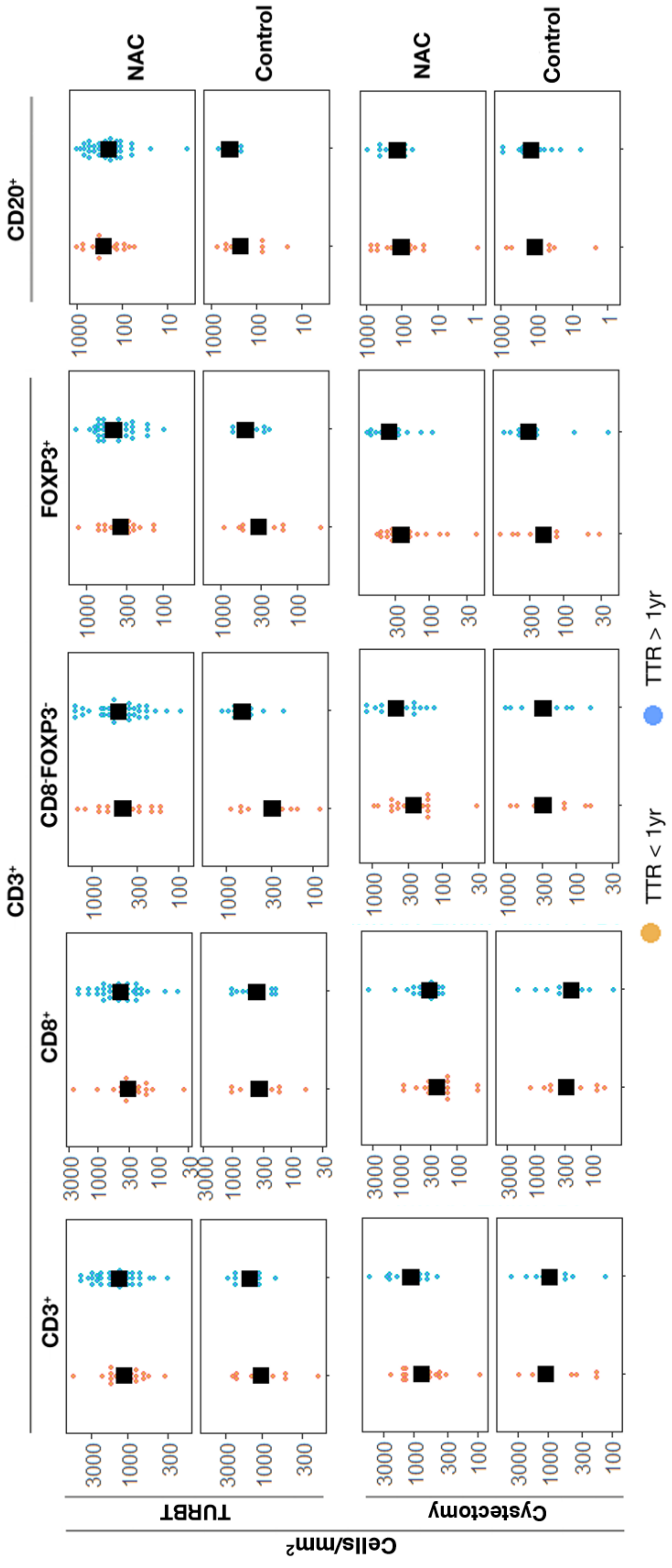

Supplementary figure 6. Prognostic value of stromal lymphocytes in TURBT and cystectomy specimens of patients with muscle-invasive bladder cancer. The plots show the absolute TIL densities with the median

depicted as a black square. Abbreviations: NAC = neoadjuvant chemotherapy, TTR = time to recurrence, TURBT = transurethral resection of the bladder tumor.

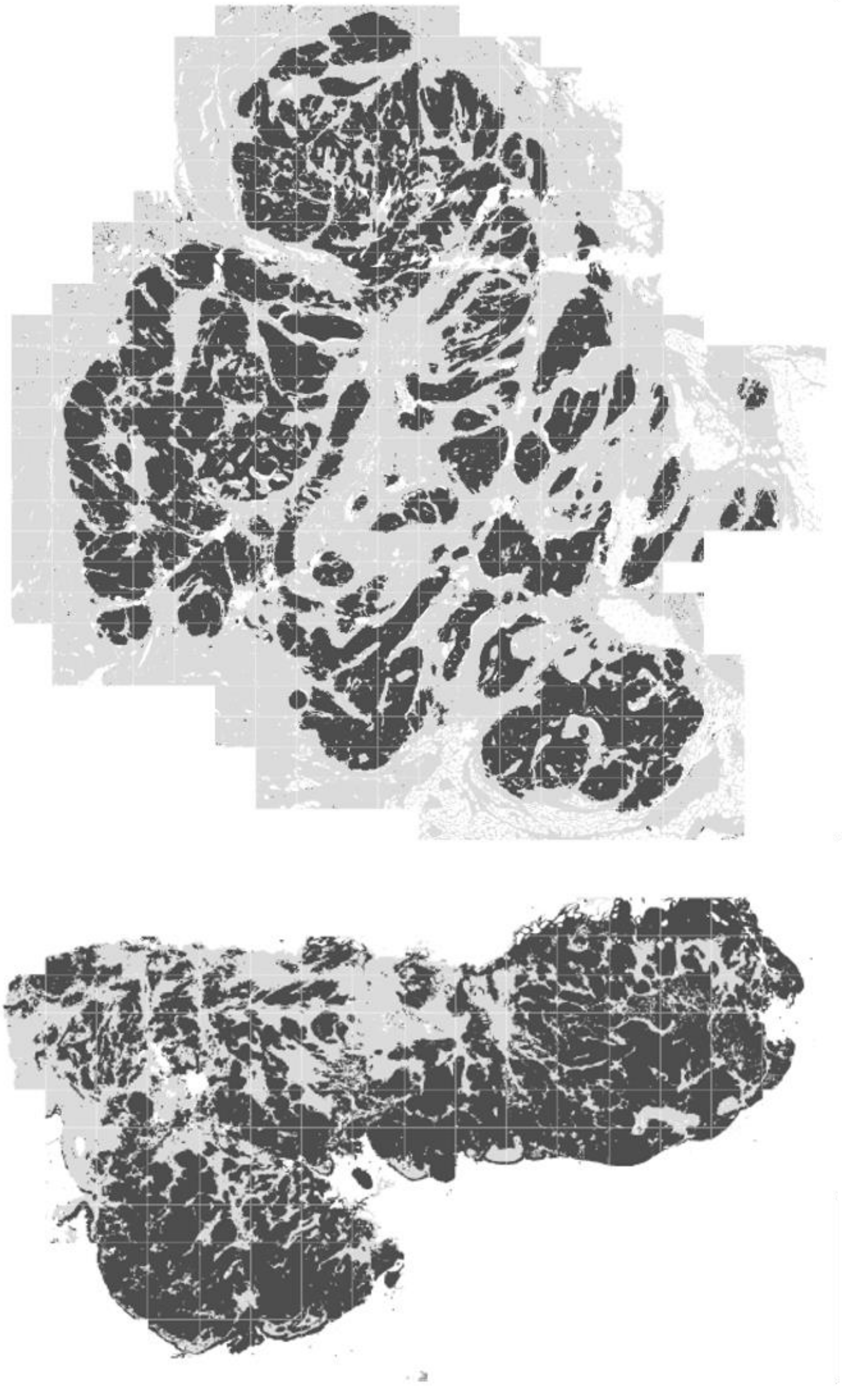

Supplementary figure 7. Tissue segmentation in a cystectomy sample and a TURBT fragment. Whereas it is reasonably possible to define an invasive margin in the cystectomy sample shown in the upper part of the figure, it is difficult to do this in the TURBT sample shown below because the original architecture is destroyed during TURBT. It is unclear whether there was more tumor at the left upper part of this TURBT fragment.

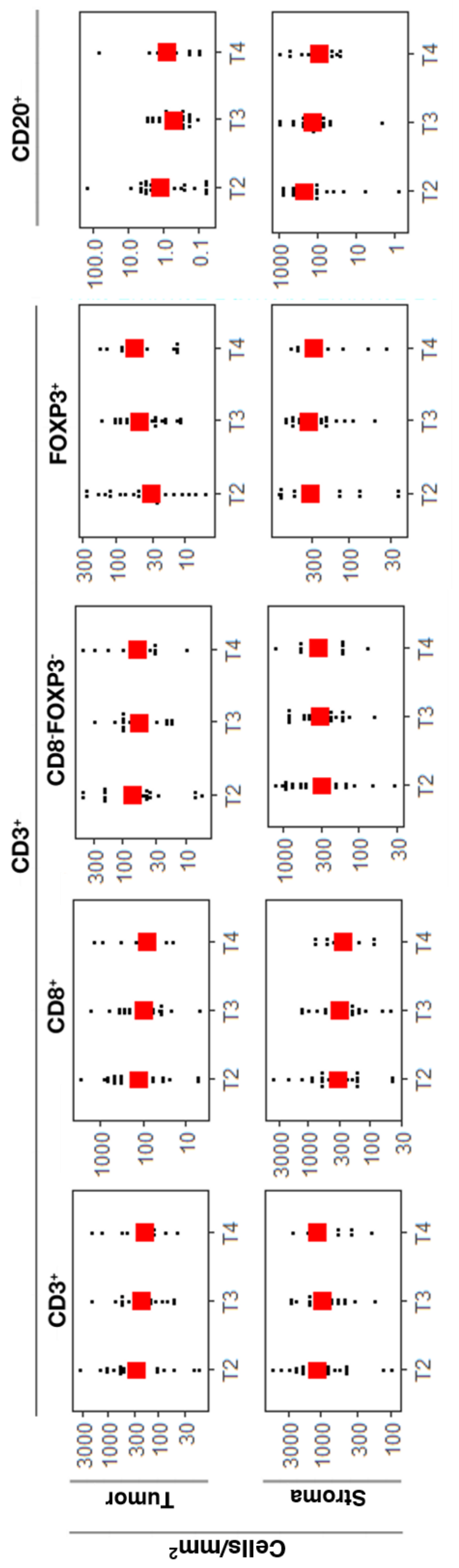

Supplementary figure 8. Differences in TIL density per T-stage in patients with muscle-invasive bladder cancer. The plots show the absolute TIL densities per T stage with the median depicted as a red square.
